# Obituary

**Published:** 1863-06

**Authors:** 


Obituary.—We are pained to hear of the death of Dr. Charles Hartman of Cleveland, Ohio, who fell upon the field of battle at Chancellorville, May 3d. Dr. Hartman was an educated, energetic and much beloved physician, and has left a family and a wide circle of friends to mourn his early and untimely death. The profession will also mourn the loss of a high-minded, intelligent, progressive physician, who was devoted to its interests and ever active in promoting its welfare.

